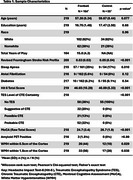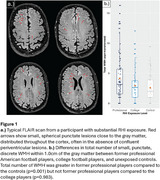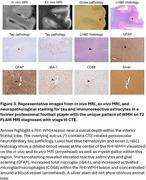# A unique pattern of T2 FLAIR white matter hyperintensities and related co‐localizing neuropathology associated with repetitive head impacts

**DOI:** 10.1002/alz70856_096739

**Published:** 2025-12-24

**Authors:** Jenna R. Groh, Annalise E. Miner, Chad William Farris, Adam Brickman, Mohamad J. Alshikho, Yorghos Tripodis, Anna Cui, Charles Adler, Laura Balcer, Charles B. Bernick, Robert C. Cantu, Michael J. Coleman, David W. Dodick, Ann C. McKee, Jesse Mez, Sylvain Bouix, Jeffrey L. Cummings, Eric M. Reiman, Martha E. Shenton, Robert A. Stern, Thor D. Stein, Michael L Alosco

**Affiliations:** ^1^ Boston University Alzheimer's Disease Research Center, Boston, MA, USA; ^2^ Boston Medical Center, Boston, MA, USA; ^3^ Columbia University, New York, NY, USA; ^4^ Boston University Chobanian & Avedisian School of Medicine, Boston, MA, USA; ^5^ Parkinson's Disease and Movement Disorders Center, Mayo Clinic, Scottsdale, AZ, USA; ^6^ NYU Langone Health, New York City, NY, USA; ^7^ Lou Ruvo Center for Brain Health, Cleveland Clinic, Las Vegas, NV, USA; ^8^ Brigham and Women's Hospital, Boston, MA, USA; ^9^ Mayo Clinic, Scottsdale, AZ, USA; ^10^ Department of Neurology, Boston University Chobanian & Avedisian School of Medicin, Boston, MA, USA; ^11^ Alzheimer's Disease Research Center, Boston University Chobanian & Avedisian School of Medicine, Boston, MA, USA, Boston, MA, USA; ^12^ Universite du Quebec, Montreal, QC, Canada; ^13^ Chambers‐Grundy Center for Transformative Neuroscience, Kirk Kerkorian School of Medicine, University of Nevada, Las Vegas, NV, USA; ^14^ Cleveland Clinic Lou Ruvo Center for Brain Health, Las Vegas, NV, USA; ^15^ Banner Sun Health Research Institute, Sun City, AZ, USA; ^16^ Brigham and Women's Hospital, Boston, MA, USA; ^17^ Boston University School of Medicine, Boston, MA, USA; ^18^ Boston University Chronic Traumatic Encephalopathy Center, Boston University Chobanian & Avedisian School of Medicine, Boston, MA, USA

## Abstract

**Background:**

Chronic traumatic encephalopathy (CTE) is a neurodegenerative tauopathy associated with repetitive head impacts (RHI). Autopsy studies show that RHI influences risk for other later‐life neuropathologies, particularly white matter and vascular injury. We have observed a unique pattern of white matter hyperintensities (WMH) on FLAIR MRI in people exposed to RHI (RHI‐WMH) characterized by small, discrete lesions near the depths of sulci (Figure 1a). The objective of this study was to characterize this pattern empirically in former American football players and to investigate neuropathology co‐localizing with the WMH.

**Method:**

The sample included 164 male former football players (*n* = 111 professional, *n* = 53 college) and 55 asymptomatic males without RHI from the DIAGNOSE CTE Project (Table 1). Visual ratings of WMH blind to the participant's RHI and clinical status were conducted to count lesions that were a.) within 1.0cm of the gray/white matter boundary, b.) as bright as the cortex, c.) spherical and d.) <1.0cm in size. ANCOVA compared groups on total number of WMH adjusted for age and vascular risk factors. We selected one individual (former professional player) with the typical RHI‐WMH pattern and who donated their brain for neuropathological examination. Guided by *in vivo* MRI, *ex vivo* MRI was done to locate the WMH near the depths of the sulci in the tissue. Tissue sections were tested for phosphorylated tau immunoreactivity and reactive astrocytes and microglia in areas surrounding the lesion seen on MRI.

**Result:**

Number of RHI‐WMH was greater in football players compared to controls (*p* = 0.007) (Figure 1b). Number of RHI‐WMH was greater in former professional players compared to controls (*p* = 0.001) but not compared to the college players (*p* = 0.983). In the neuropathology case study, we located the punctate RHI‐WMH on *ex vivo* MRI. Immunostaining revealed that the RHI‐WMH was adjacent to a sulcus with the pathognomonic CTE *p*‐tau lesion and was characterized by myelin pallor, dense GFAP‐positive glial scarring, and increased total and activated microglia (Figure 2).

**Conclusion:**

We provide empirical support for exposure to RHI as potentially leading to a unique pattern of WMH. Importantly, histopathological evidence showed that the RHI‐WMH co‐localized with RHI and CTE‐related neuropathologies at the depths of the sulci.